# Massive retroperitoneal cyst impersonating ovarian tumor: A case report

**DOI:** 10.1016/j.ijscr.2022.107393

**Published:** 2022-07-12

**Authors:** Hidar Alibrahim, Amany Al Ali, Haidara Bohsas, Safaa Mohamed Alsharief Ahmed, Eman Mohammed Sharif Ahmed, Sarya Swed

**Affiliations:** a Faculty of Medicine, Aleppo University, Aleppo, Syria; b Faculty of Medicine, Hama University, Hama, Syria; c Shendi University, Sudan; d Nile Valley University, Sudan

**Keywords:** Massive retroperitoneal cyst, Ovarian tumor, Case report

## Abstract

**Introduction:**

The retroperitoneum is the anatomical compartment positioned behind the peritoneal cavity. It is separated into three primary spaces: the anterior pararenal, perirenal, and posterior pararenal spaces. Retroperitoneal cystic mass is a rare surgical problem that is often wrongly identified before surgery.

**Case presentation:**

We report a case of a 27-year-old female presenting with abdominal swelling and pain starting from 9 months. An abdominal computed tomography scan showed a right adnexal mass with a high probability of a serous ovarian. The patient was diagnosed with ovarian tumors before surgery, but it was identified with a retroperitoneal cyst during surgery.

**Discussion:**

A retroperitoneal cyst's clinical signs and symptoms vary, and the diagnosis can often be challenging. Computed tomography scans are appropriate for assessing retroperitoneal pathology because they produce separate sectional images and couldn't find the correct diagnosis in previous cases.

**Conclusion:**

This paper shows the rare case of primary retroperitoneal lesions, which can be hard to diagnose before surgery, even though medical imaging has come a long way.

## Highlights

• A retroperitoneal cystic tumor is a very uncommon medical condition that is often misdiagnosed prior to surgery.
• The diagnosis is made based on the patient's medical history as well as imaging using a computed tomography (CT) scan, which shows unilocular or multilocular thin-walled cyst lesions with watery liquids.
• Although a CT scan can aid in the detection of these lesions, surgery is still the essential factor in identifying the diagnosis.
• Clinical doctors should be aware in conducting early accurate diagnosis when facing like these lesions to avoid severe complications.

## 1 Introduction

The retroperitoneal is the anatomical region characterized by the transversal fascia and the parietal peritoneum. It consists of three primary divisions, the anterior pararenal space, perirenal space, and posterior pararenal space. It contains the abdominal organs such as the kidneys, adrenal glands, ureters, ascending colon, descending colon, the duodenum, pancreas, and the abdominal aorta [1], [2]. Various lesions can develop in the retroperitoneum, with most malignant lesions such as lymphoma, liposarcoma, lymphangioma, and cystadenoma. Furthermore, retroperitoneal cystic lesions are classified as malignant or benign, and the most prevalent types of benign retroperitoneal lesions are lymphangioma and cystic mesothelioma [3], [4]. Cystic mesothelioma is a rare benign tumor, with predominance in women, and it may relapse locally [4]. The most common symptom of retroperitoneal cystic mesothelioma is abdominal pain, and in rare cases, patients may present with dysfunctional uterine bleeding, early satiety, urinary urgency, and dyspareunia [5]. The diagnosis depends on the clinical history and imaging with computed tomography scan, which appears as unilocular or multilocular thin-walled cyst lesions containing watery liquids [4], [5]. Every year, ovarian cancer affects 239,000 people worldwide and causes 152,000 deaths [6]. According to the WHO, ovarian cancer is classified into numerous morphological subtypes, including serous carcinomas, endometrioid carcinomas, mucinous carcinomas, clear-cell carcinomas, transitional-cell Brenner tumors, and the undifferentiated type [7]. Despite that, ovarian cancer symptoms have always been ambiguous and unclear, ranging from abdominal bloating, urinary frequency, abdominal pain, early satiety, and changes in bowel habits [8]. After the medical history and symptoms, the Doppler transvaginal ultrasonography is the first assessment method for ovarian cancer, which reveals the malignancy manifestations as complexity with solid and cystic areas, extramural fluid, echogenicity, wall thickening, septa, and papillary projections with a greater number of arteries, and the gadolinium-enhanced magnetic resonance imaging can be used for further assessment. However, benign masses typically manifest as sonolucent masses with smooth walls [9].

Here we reported a case of a 27-year-old female with a huge retroperitoneal cyst masquerading as an ovarian tumor. We recommended that clinical doctors be aware of retroperitoneal mass when there are imaging features of ovarian tumors.

The case was written by following the scare checklist guidelines 2020 for writing case reports [16].

## 2 Case presentation

**Fig. 1:**
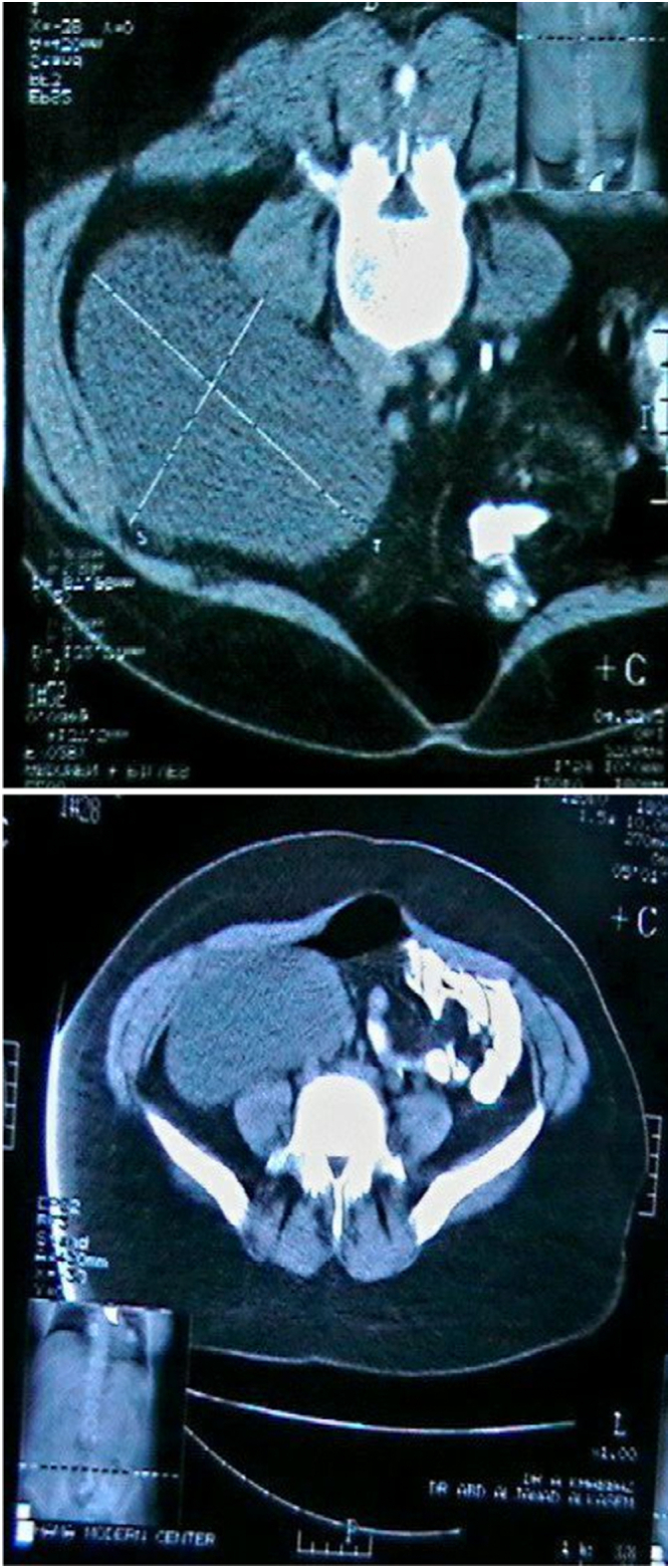
A computed tomography (CT) scan of the abdomen showed a right adnexal mass that was highly probable a serous ovarian tumor.

**Fig. 2:**
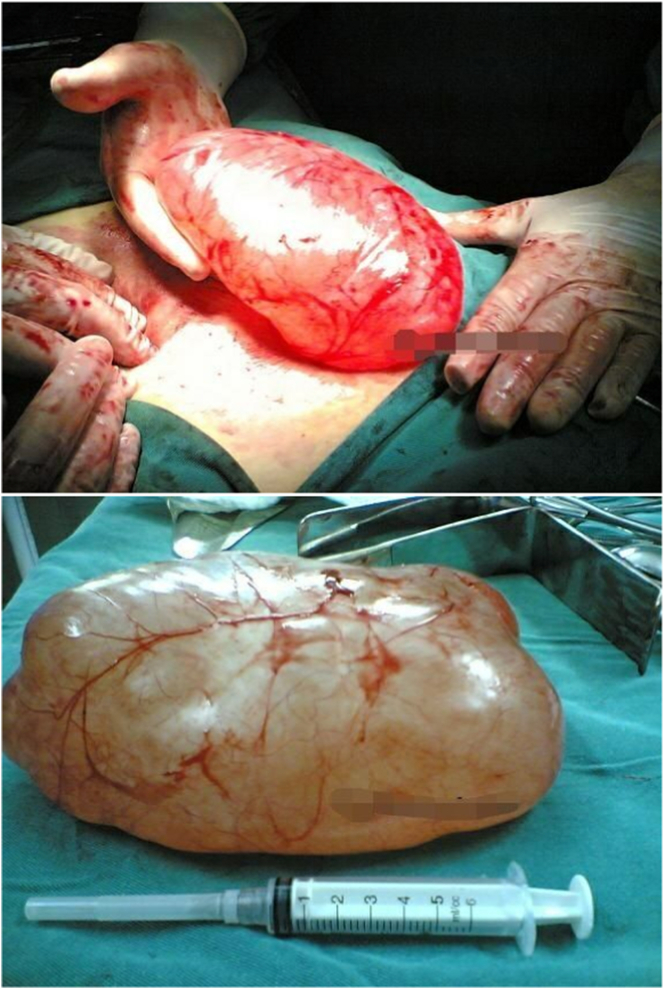
The pictures show the mass that was removed.

A 27-year-old female presented to the University Hospital complaining of abdominal swelling and abdominal pain starting from 9 months and five months, respectively, with heaviness in the abdomen. Her medical history was clear, and the physical examination revealed a right flank abdominal pain extending to the right iliac fossa, with a palpable mass in the right side of the abdomen extending to the pelvis, with no hepatosplenomegaly or palpable lymph nodes. Complete blood tests were within normal range, and there were no tumor markers tests due to insufficient financial capabilities. Abdominal ultrasound revealed a multiloculated cyst with an incomplete thick septum across the right side of the abdomen, with no solid component. Abdominal computed tomography showed a mass extended to the right side of the abdomen, so we suggested it may be a right adnexal mass with a high probability of a serous ovarian tumor measuring approximately (13 × 10 × 15 cm) (Fig. 1), although Doppler transvaginal ultrasonography was an unremarkable. After that, the patient was prepared for the surgical approach. A laparotomy revealed a massive cyst that developed from the retroperitoneal region (Fig. 2), and the retroperitoneal cyst was resected without any complications. The histopathological result was a benign retroperitoneal cyst. After following up for two weeks, her general condition improved, and she was discharged from the hospital. After three months of the surgical approach, the abdominal ultrasound revealed no signs of recurrence.

## 3 Discussion

A retroperitoneal cyst's clinical signs and symptoms vary, and the diagnosis can often be challenging [10]. These masses may grow to huge sizes before pressuring nearby structures and causing symptoms; alternatively, they may be discovered during diagnostic imaging for another reason [11]. Retroperitoneal cysts (RPCs) are divided into groups according to their embryologic genesis and histological differentiation. Retroperitoneal cysts (RPCs) are divided into groups according on their embryologic genesis and histological differentiation [12]: (a): Urogenital; (b): mesocolic; (c): cysts arising in cell inclusions; (d): traumatic; (e): parasitic and (f): lymphatic. This paper showed a 27-year-old female presenting with abdominal swelling and pain starting from 9 months. Physical examination revealed a right flank abdominal pain extending to the right iliac fossa, with a palpable mass in the right side of the abdomen extending to the pelvis. Abdominal computed tomography showed a right adnexal mass with a high probability of a serous ovarian. The patient was diagnosed with ovarian tumors before surgery, and a laparotomy revealed a massive cyst that developed from the retroperitoneal region. By comparing our case with similar documented cases in the literature, we found Syamim Johan et al. [13], a patient with abdominal swelling for a 1-year duration. Clinically, the abdomen was soft and non-tender, with a mass palpable over the left lumbar area. The abdominal radiograph showed a huge radiopaque lesion over the left abdomen.

Computed tomography scan showed left adnexal mass with a possibility of ovarian serous cystadenoma measuring 14.5 × 14.6 × 18.2 cm with local mass effect to the ureter causing mild left hydronephrosis. Intraoperatively, a vast mass arose from the retroperitoneal space attaching to the psoas muscle, spine, and encasing the internal iliac and left ureter. Rafique U Harvitkar et al. [14] a patient with no comorbidities was referred due to incidental discovery of a voluminous retroperitoneal pelvic cyst during diagnostic laparoscopy .abdomen examination; she was found to have a soft lax abdomen, central obesity, and no palpable lump. An ultrasonography (USG) scan of the abdomen revealed a large (12.4 × 10.2 × 10 cm) multiloculated, thick-walled cystic mass with low internal echoes noted posterior and left to the uterus separate from both the ovaries, suggesting hydrosalpinx with mild hypervascularity. A magnetic resonance imaging (MRI) scan of the pelvis confirmed the USG findings. At laparoscopy, a large retroperitoneal multiloculated cystic mass measuring 12 × 10 cm was noted more towards the left of the midline next to the left rectal wall. Ultrasound is an important investigative tool that assists in making more realistic and practical decisions. However, it has a limited usefulness in identifying intra-abdominal abnormalities, especially retroperitoneal abnormalities. CT scans are appropriate for assessing retroperitoneal pathology because they produce separate sectional images but also, they couldn't find the correct diagnosis in previous cases. However, the absence of internal echoes helps differentiate them from abscesses, hematomas, and complicated cysts such as dermoid or hydatids; also, they can be distinguished from lymphoceles or urinomas, which commonly develop after surgery or after an attack of ureteric obstruction [15].

Because they continue to recur following simple aspiration, retroperitoneal cysts should be surgically removed. In this article, we showed a rare case that caused a diagnostic challenge due to overlapping imaging findings, where only by surgery we discovered the correct diagnosis.

## 4 Conclusion

Cysts originating outside the major organs of the retroperitoneum are incredibly uncommon. Although a CT scan can aid in the detection of these lesions, surgery is still the essential factor in identifying the diagnosis. This article aims to raise awareness among doctors about retroperitoneal cysts, especially when they are masquerading as ovarian tumors.

## Consent for publication

Written informed consent was obtained from the patient for publication of this case report and accompanying images. A copy of the written consent is available for review by the Editor-in-Chief of this journal.

## Provenance and peer review

Not commissioned, externally peer reviewed.

## Ethical approval

This case report didn't require review by Ethics committee, Aleppo university hospital, Aleppo University, Aleppo-Syria.

## Funding

This research did not receive any specific grant from funding agencies in the public, commercial, or not-for-profit sectors.

## Guarantor

Hidar Alibrahim.

## Research registration number

Not applicable.

## CRediT authorship contribution statement

Hidar Alibrahim: contributed in writing manuscript and data collecting, Amany Al Ali: contributed in writing manuscript, Haidara Bohsas: contributed in writing manuscript, Safaa Mohamed Alsharief Ahmed: contributed in writing manuscript, Eman Mohammed Sharif Ahmed: contributed in writing manuscript, Sarya Swed: contributed in reviewing the paper.

## Declaration of competing interest

All authors declared no conflict of interest.

## Appendix A Supplementary data

Supplementary material Image 1

## References

[1] Coffin A., Boulay-Coletta I., Sebbag-Sfez D., Zins M. (2015). Radioanatomy of the retroperitoneal space. Diagn. Interv. Imaging.

[2] Lambert G., Samra N.S., StatPearls (2022). Retroperitoneum.

[3] Yacoub J.H., Clark J.A., Paal E.E., Manning M.A. (2021). Approach to cystic lesions in the abdomen and pelvis, with radiologic-pathologic correlation. Radiographics.

[4] Yang D.M., Jung D.H., Kim H., Kang J.H., Kim S.H., Kim J.H., Hwang H.Y. (2004). Retroperitoneal cystic masses: CT, clinical, and pathologic findings and literature review. Radiographics.

[5] O'Neil J.D., Ros P.R., Storm B.L., Buck J.L., Wilkinson E.J. (1989). Cystic mesothelioma of the peritoneum. Radiology.

[6] Reid B.M., Permuth J.B., TAJCb Sellers (2017). Epidemiology of ovarian cancer: a review. Medicine.

[7] Young R. (2014).

[8] Stewart C., Ralyea C., Lockwood S. (2019). Ovarian cancer: an integrated review. Semin. Oncol. Nurs..

[9] Roett M.A., Evans P. (2009). Ovarian cancer: an overview. Am. Fam. Physician.

[10] Dunev V.R., Genov P.P., Kirilov I.V., Mladenov V.D. (2021). Retroperitoneal cystic lymphangioma-a case report. Urol. Case Rep..

[11] Sharma G., Shreshtha S., Bhatt S., Garg P.K. (2019). Retroperitoneal cystic mass: a diagnostic challenge. ANZ J. Surg..

[12] Alzaraa A., Mousa H., Dickens P., Allen J., Benhamida A. (2008). Idiopathic benign retroperitoneal cyst: a case report. J. Med. Case Rep..

[13] Johan S., Hassan M.F., Hayati F., Azizan N., Payus A.O., Edwin See U.H. (2020). Huge retroperitoneal cyst masquerading as ovarian tumour: a case report. Front. Surg..

[14] Harvitkar R.U., Sankpal R., Joshi A. (2021). Huge pelvic retroperitoneal cyst masquerading as hydrosalpinx: a case report with review of the literature. J. Family Med. Prim. Care.

[15] Bradley M.J., Cosgrove D.O., Allan P.L., Baxter G.M., Weston M.J. (2011). Clinical Ultrasound.

[16] Agha R.A., Franchi T., Sohrabi C., Mathew G., for the SCARE Group (2020). The SCARE 2020 guideline: updating consensus Surgical CAse REport (SCARE) guidelines. International Journal of Surgery.

